# Exploring feasibility, perceptions of acceptability, and potential benefits of an 8-week yoga intervention delivered by videoconference for young adults affected by cancer: a single-arm hybrid effectiveness-implementation pilot study

**DOI:** 10.1186/s40814-023-01244-y

**Published:** 2023-03-10

**Authors:** Amanda Wurz, Emma McLaughlin, Kimberly Hughes, Kelsey Ellis, Amy Chen, Lauren Cowley, Heather Molina, Delaney Duchek, Maximilian Eisele, S. Nicole Culos-Reed

**Affiliations:** 1 School of Kinesiology, University of the Fraser Valley, Chilliwack, Canada; 2 Faculty of Kinesiology, University of Calgary, Calgary, Canada; 3 Orenda Society, Calgary, Canada; 4 City University, Vancouver, Canada; 5 Department of Oncology, Cumming School of Medicine, University of Calgary, Calgary, Canada; 6 Department of Psychosocial Resources, Tom Baker Cancer Centre, Cancer Care, Alberta Health Services, Calgary, Canada

**Keywords:** Movement, Physical activity, Exercise, Oncology, Pragmatic approach

## Abstract

**Background:**

Young adults affected by cancer face physical and psychological challenges and desire online supportive care. Yoga can be delivered online and may improve physical and psychological outcomes. Yet, yoga has rarely been studied with young adults affected by cancer. To address this, an 8-week yoga intervention was developed, and a pilot study was deemed necessary to explore feasibility, acceptability, implementation, and potential benefits.

**Methods:**

A mixed-methods, single-arm hybrid effectiveness-implementation pilot study evaluating the yoga intervention was conducted. Feasibility was assessed by tracking enrollment, retention, attendance, completeness of data, and adverse events. Acceptability was explored through interviews. Implementation metrics included training time, delivery resources, and fidelity. Potential effectiveness was evaluated by exploring changes in physical (i.e., balance, flexibility, range of motion, functional mobility) and psychological (i.e., quality of life, fatigue, resilience, posttraumatic growth, body image, mindfulness, perceived stress) outcomes at pre- (week 0), post- (week 8), and follow-up (week 16) time points. Data were analyzed with descriptive statistics, repeated measures analysis of variance, and content analysis.

**Results:**

Thirty young adults participated in this study (recruitment rate = 33%). Retention to study procedures was 70%, and attendance ranged from 38 to 100%. There were little missing data (< 5%) and no adverse events. Though most participants were satisfied with the yoga intervention, recommendations for improvement were shared. Sixty study-specific training hours and > 240 delivery and assessment hours were accrued and fidelity was high. Functional mobility, flexibility, quality of life (energy/fatigue, social well-being), body image (appearance evaluation), mindfulness (non-reactivity), and perceived stress improved significantly over time (all *p*< 0.050; $\eta_{p}{}^{2}s=0.124-0.292$). No other significant changes were observed (all *p*> 0.050; $\eta_{p}{}^{2}s=0.005-0.115$).

**Conclusions:**

The yoga intervention may confer physical and psychological benefits, though intervention and study-specific modifications are required to improve feasibility and acceptability. Requiring study participation and providing greater scheduling flexibility could enhance recruitment and retention. Increasing the frequency of classes offered each week and offering more opportunities for participant interaction could improve satisfaction. This study highlights the value of doing pilot work and provides data that has directly informed intervention and study modifications. Findings could also be used by others offering yoga or supportive care by videoconference to young adults affected by cancer.

**Trial registration:**

Not available—not registered

**Supplementary Information:**

The online version contains supplementary material available at 10.1186/s40814-023-01244-y.

## Key messages regarding feasibility:

- What uncertainties existed regarding the feasibility?
  - Prior to this pilot study, the feasibility of yoga delivered via videoconference to young adults affected by cancer was unknown.
- What are the key feasibility findings?
  - The yoga intervention and study were partially feasible; recruitment and retention to the study were lower than expected, but for those completing the study attendance rates were high, missing data was low, and there were no adverse events.
- What are the implications of the feasibility findings for the design of the main study?
  - Modifying recruitment procedures and offering greater scheduling flexibility could enhance aspects of feasibility that were lower than expected.

## Background

Young adults diagnosed with cancer between the ages of 18 and 39 years represent roughly 5% of all new cancer diagnoses each year in North America [1, 2]. Though comprising a minority of new diagnoses, this population faces a greater number of life years lost and life years affected by morbidity compared to the general population [3, 4]. Furthermore, many young adults affected by cancer experience adverse and interrelated physical (e.g., weight gain/loss, cachexia, muscle loss, disfigurement) [5–7] and psychological effects (e.g., reduced self-esteem, negative perceptions of body image, lowered quality of life, anxiety) [8–10] and describe feeling lonely and isolated [11, 12]. Group-based interventions or programs that can positively impact physical and psychological outcomes are required for this population.

Yoga, as practiced in Western societies, typically includes physical postures, mindfulness/meditation, and breathwork. Among older adults with a history of cancer, findings from systematic reviews and meta-analyses suggest yoga can enhance physical (e.g., flexibility, range of motion) and psychological outcomes (e.g., symptoms of anxiety, negative affect, quality of life) [13–15]. Among young adults affected by cancer, results from an experimental [16] and cross-sectional study [17] suggest that yoga is desired and may offer similar benefits to those seen among older adults with a history of cancer. Nevertheless, few yoga interventions have been studied, programs in clinical or community settings developed for young adults affected by cancer are rare, and the potential range of benefits yoga may confer for this population remains relatively unexplored.

One reason for limited yoga interventions and programs for young adults affected by cancer may be related to their unique barriers to participation (e.g., small and spread out population, difficulty coordinating schedules amidst conflicting life/work demands, incidental costs to participation) [18, 19]. Telehealth modalities, such as videoconferencing, could address many of these barriers and is a preferred delivery style for interventions among young adults affected by cancer [20, 21]. Indeed, the feasibility and effectiveness of delivering interventions, such as mindfulness and self-compassion, via videoconference within this cohort have been demonstrated [22, 23]. Furthermore, there is experimental evidence showing the benefits of adapted yoga [24] and physical activity [25] delivered by videoconference to adults affected by cancer > 40 years of age.

Studies exploring the effectiveness and implementation of yoga when delivered via videoconference could lay a foundation for future yoga research as well as the development and implementation of programs. Hybrid effectiveness-implementation studies are one way to evaluate interventions and their implementation strategies [26]. However, before proceeding with a full-scale hybrid effectiveness-implementation study, a pilot study is warranted. A pilot study can offer invaluable insights into the feasibility and acceptability of an intervention prior to expending resources on a full-scale study [27]. Thus, the aims of this single-arm hybrid effectiveness-implementation pilot study were to (1) assess feasibility (i.e., recruitment to the study, retention, attendance, adverse events, completion of assessments) and acceptability (i.e., satisfaction) with the yoga intervention and study methods; (2) document markers of implementation (i.e., training time, delivery resources, fidelity); and (3) explore preliminary effectiveness of the yoga intervention via changes over time in physical (i.e., balance, range of motion, flexibility, functional mobility) and psychological (i.e., quality of life, fatigue, resilience, posttraumatic growth, body image, mindfulness, perceived stress) outcomes.

## Methods

### Study design

A single-arm hybrid effectiveness-implementation pilot study was conducted, and a mixed-methods, embedded approach was used wherein both quantitative and qualitative data were collected [28]. The study protocol was reviewed and approved by the Health Research Ethics Board of Alberta (HREBA.CC-20-0365). To enhance transparency in reporting, relevant aspects of the Consolidated Standards of Reporting Trials extension for pilot and feasibility trials (CONSORT [29]), the Standards for Reporting Implementation Studies (StaRI) statement [30], and the CheckList stAndardising the Reporting of Interventions For Yoga (CLARIFY) guidelines [31] were followed in the preparation of this manuscript (see Additional file 1).

### Participants

Young adults affected by cancer from across Canada were eligible to take part in the study if they (1) were diagnosed with cancer between the ages of 18 and 39 years; (2) were at any stage of the cancer trajectory (i.e., diagnosis onward); (3) self-reported being able to participate in mild-to-moderate intensity yoga delivered online; (4) registered to participate in the online intervention in the fall 2020 or winter 2021 wave; and (5) were able to read, understand, and provide informed consent in English.

### Procedures

Young adults affected by cancer were recruited to participate in the yoga intervention through email, social media (i.e., Facebook, Twitter, Instagram), and snowball sampling. After registering to take part in the yoga intervention, young adults were then invited to take part in the pilot study to evaluate the intervention^1^. For those who were interested and provided informed consented, assessments were conducted at baseline (week 0), post-intervention (week 8), and follow-up (week 16). The assessments comprised virtually administered physical assessments using videoconference (i.e., Zoom), and an online survey housed on REDCap. Also, post-intervention (week 8) participants completed an interview via videoconference.

### Yoga intervention

The yoga intervention was informed by Yoga Thrive, an evidence-based therapeutic yoga program for individuals affected by cancer and their support persons, and the expertise of the study team. Findings from recent systematic reviews [13–15], studies describing young adults’ virtual delivery preferences [20, 21], evidence-based behavior change techniques [32, 33], and perspectives from 12 young adults affected by cancer who participated in online pilot yoga classes at the start of the pandemic were also incorporated into the design of the intervention. The result was a yoga intervention for young adults affected by cancer and their support persons^2^ delivered over an 8-week period, with one, 60-min class offered per week. In the fall 2020 wave, two different sessions were offered and in the winter 2021 wave, three different sessions were provided. In each wave, young adults could register in the class day/time that best suited their schedule.^3^ Each class in the intervention had a specific theme comprising a physical focus and energetic intention (e.g., *chest opening and shoulder mobility* and *practicing gratitude*) and included physical postures, breath practices, and meditation/relaxation techniques. Also, behavior change support was provided throughout via autonomy-supportive instruction, social support, and journaling and reflection prompts. Throughout classes, participants were offered various modifications (including seated postures), were reminded of common contraindications and provided alternative poses, and were continuously supported in choosing postures that felt appropriate and comfortable for them. See Table 1 for a general sequence of a class within the intervention. The entire 8-week protocol is available, upon reasonable request, from the first author.

**Table 1:** General sequence of a class within the 8-week yoga intervention

Class component	Example prompts, practices, and postures
Greeting	Open-ended questions to group to answer via chat: *How are you? What is your energy like today? How are you feeling?*
Supine (or seated)	Breathwork • Natural breath • Even counted breath • Box breath Postures • Modified wind-relieving pose • Supported spinal twist • Lateral flexion • Legs up the wall • Core activation • Bridge
Seated or kneeling	Breathwork • Natural breath • Joining breath to movement Postures • Seated forward fold • Supported spinal twist • Lateral flexion • Cat and cow • Child’s pose • Arm/leg extension • Seated tree pose
Standing	Breathwork • Natural breath • Joining breath to movement Postures • Tree pose • High lunge • Warrior II • Half march • Mountain • Chair
Seated or kneeling	Breathwork • Natural breath • Joining breath to movement Postures • Seated forward fold • Supported spinal twist • Lateral flexion • Dear pose • Seated figure 4 • Neck release
Supine (or seated)	Breathwork • Natural breath • Even counted breath • Box breath Postures • Savasana (corpse pose) • Comfortable seat Mindfulness • Body scan • Visualization • Reading
Journaling and reflection	Open-ended questions to the group to reflect on and journal about quietly: *What does self-care mean to me? How am I disappointing myself to avoid disappointing others? What is one thing I can do more/less of that would be an act of self-care?*
Closing, check-out, and reflection	Open-ended questions for the group to answer via chat: *How are you feeling now? What did the journal prompts bring up for you?*

*Note*: Every class in the 8-week session differed, including the length of time spent in each component. Prompts, practices, and postures aligned with the physical focus and energetic intention

Classes were led by one of three yoga instructors who had completed at least a 200-h yoga teacher training, Thrive Health Exercise Oncology training, Yoga Thrive Teacher Training Certification (or similar), and had practical experience delivering yoga to individuals affected by cancer. Classes were also moderated by two individuals with Thrive Health Exercise Oncology training and practical experience moderating physical activity classes for adults affected by cancer. Moderators welcomed participants to each class, communicated with participants between classes via email, fostered a positive social environment, notified the instructor if/when further instruction or details were needed for a posture, demonstrated seated postures (from a chair in classes with higher risk participants), and managed the chat feature in Zoom during classes.

### Measures

#### Feasibility and acceptability

##### Feasibility

Throughout the study, to assess feasibility, the number of young adults recruited to the study from the yoga intervention, retention to study procedures, and attendance to the yoga classes were collected. Completion of physical assessments, online surveys, and interview were also tracked, and adverse events were recorded. Recruitment to the study was defined as the number of young adults who enrolled in the study out of the number of young adults who were registered in the yoga intervention. Retention rate was defined as the number of study participants who completed all three assessments within the specified time frame (i.e., within 2 weeks). Attendance was defined as the number of classes engaged in out of eight for those in the yoga study. Completeness of physical assessments, online surveys, and participation in interviews was examined, and the percentage of missing data for each was tracked. Adverse events were defined as any incident causing harm to the participant. Participants were instructed to self-report adverse events, and moderators were trained to report any adverse events occurring during yoga classes using a standardized reporting form (e.g., date, severity, timing, site/location, duration, clinical action taken, outcome).

Aligned with recommendations for conducting pilot studies [34], targets for each feasibility outcome were specified a priori using relevant literature. It was estimated that (1) there would be a 60% recruitment rate from the intervention to the study (i.e., 55 young adults would be recruited to the study) [22, 23]; (2) ≥ 75% of young adults in the study would complete baseline (week 0), post-intervention (week 8), and follow-up (week 16) assessments [35]; (3) > 75% of participants would attend ≥ six of eight yoga classes [22, 23]; and (5) there would be < 10% missing data [36].

##### Acceptability

Post-intervention (week 8), participants answered questions during a semi-structured interview (see the “Interviews” section) related to their satisfaction with the yoga intervention (e.g., delivery, modality, length, duration, group-based nature) and study methods (e.g., satisfaction with assessments, procedures). The questions asked during the interview can be found in Additional file 2.

#### Implementation

Throughout the study, all aspects related to the delivery of the yoga intervention, including training time, delivery resources, and fidelity were tracked. Training time was defined as the number of hours required to train moderators on the study protocol (e.g., class facilitation, adverse event responding and reporting, virtual physical assessment conduct) and yoga instructors on the yoga intervention. Delivery resources captured the number of hours of intervention delivery, personnel hours (moderating, instructing, and/or completing physical assessments and interviews), and administrative support (i.e., the time required for intervention outreach, study recruitment, potential communication with prospective and current participants [e.g., responding to queries, coordinating physical assessments and interviews, and sending weekly reminder emails to participants]). Finally, fidelity was defined as whether the intervention was delivered as intended or not. Fidelity was tracked across classes by moderators using a standardized form to document instructors’ greetings and closing remarks, offering modifications, and use of autonomy-supportive language. Also, immediately after each class, yoga instructors completed an additional fidelity checklist to indicate any deviations to the class plan (i.e., additions or omissions of postures/poses).

#### Potential effects of yoga

##### Personal and medical factors

At baseline (week 0), participants in the study self-reported their age, location (i.e., province), setting (i.e., rural/urban), biological sex, current gender, marital status, education, annual income, employment status, ethnicity, cancer diagnosis, treatment status, and symptoms.

##### Physical outcomes

At baseline (week 0), post-intervention (week 8), and follow-up (week 16), participants completed a battery of physical assessments delivered over Zoom by one of three trained assessors who had previous experience administering these tests in-person and online to older adults affected by cancer. Neither assessors nor participants were blinded, which is common in pragmatic and behavioral studies [37, 38]. Assessments included *balance* using the single-leg balance test [39], *flexibility* using sit-and-reach [40], *shoulder range of motion* using the shoulder flexion test [41], and *functional mobility* via the 30-s sit-to-stand [42]. A more detailed description of the scoring for each of these assessments can be found in Additional file 3.

##### Psychological outcomes

At baseline (week 0), post-intervention (week 8), and follow-up (week 16), participants completed an online survey, housed in REDCap, comprised of measures of *quality of life* using the RAND 36-Item Short Form Health Survey [43], *fatigue* using the FACIT-Fatigue Scale [44], *resilience* using the Brief Resilience Scale [45], *sense of personal growth* after cancer with the Posttraumatic Growth Inventory [46], *body image* using the Multidimensional Body-Self Relations Questionnaire Appearance Scales [47], *mindfulness* using the Five-Facet Mindfulness Questionnaire [48], and *perceived stress* with the Perceived Stress Scale [49]. Also, *connection to the yoga group* was assessed with a modified version of the Group Identification Scale [50] at post-intervention (week 8) only. Further details related to the scoring each of these questionnaires can be found in Additional file 4.

##### Interviews

Post-intervention (week 8), participants completed interviews following a semi-structured guide, with one of two trained study team members. Participants were asked a series of open-ended questions (with probes) covering acceptability (see the “Acceptability” section). All interviews were conducted via Zoom and were audio-recorded using a Sony ICD-PX240 recorder. During the interviews, questions situating yoga within participant’s cancer experience and exploring additional important outcomes were also collected. These data are not presented herein given the scope of this pilot study reporting on feasibility, acceptability, implementation, and potential effectiveness.

### Sample size

No formal sample size calculation was performed based on the study objectives.

### Data analysis

Quantitative data were analyzed using IBM SPSS (version 27). Descriptive statistics (i.e., means, standard deviations [SD], frequencies, percentages) were computed to describe the sample at baseline. Following this, data were checked for approximately normal distribution^4^, univariate (*z*-score greater than 3 or less than − 3) and multivariate outliers (*p*-value < 0.01 on the Mahalanobis Distance Test), and sphericity. In cases where outliers were identified, sensitivity testing was performed (with and without outliers) to affirm consistent trends in the data and then outliers were removed on a variable-by-variable basis to enhance homogeneity and maximize statistical power. Repeated measures analysis of variance [51] were conducted to examine changes across time points (baseline [week 0], post-intervention [week 8], follow-up [week 16]) in physical and psychological outcomes. Of note, data were not nested based on wave or instructor, no adjustments were made, and a higher type I error probability was set (i.e., an uncorrected significance level of 0.05) to decrease the risk of missing a potentially beneficial effect of yoga^5^. The effect size of these changes was computed with partial eta squared ($\eta_{p}{}^{2}$; small effect = 0.01, medium effect = 0.06, large effect = 0.14).

To analyze the qualitative data, interviews were transcribed verbatim and uploaded into NVivo (version 12) where they were subsequently analyzed by one author (EM) using conventional content analysis [52]. First, EM read each transcript several times to immerse herself in the data. Next, EM coded transcripts, created labels reflecting key ideas, and sorted the codes into higher-order categories. At this point, the author sent the coding scheme to another author (AW) who had reviewed the transcripts several times and challenged EM’s thoughts and interpretations. Following this, EM generated definitions for each category and selected exemplar quotes from the data to illustrate findings from the interviews. The penultimate coding scheme was then sent to all authors, each of whom was involved in the study design, intervention delivery, and/or data collection, to review and approve. Following this, EM revisited all raw data to ensure participants voices were accurately represented and the coding scheme was finalized. To promote rigor and trustworthiness, several steps recommended in the literature were followed [53]. The two authors who conducted the interviews (EM, KE) and one author who conducted the content analysis (EM) kept reflexivity journals and continuously (re-)examined their own perspectives and how they might influence interpretations. A critical friend (AW) challenged interpretations and sought to ensure the results represented participants’ voices and all authors critically reviewed the findings, and finally, category descriptions and exemplar quotes are available and presented herein to provide transparency.

## Results

### Participants

As detailed in Table 2, study participants were on average 34.2 (SD = 5.09) years of age at baseline and most self-identified their biological sex as female (*n* = 28; 93%) and as being of Western European descent (*n* = 17; 57%). Participants reported having been diagnosed with cancer between 2012 and 2020, and nearly half were diagnosed with breast cancer (*n* = 13; 43%). Fourteen (47%) reported being on-treatment at the time of the study, and the remainder were off-treatment (*n* = 13; 43%) or did not report their treatment status (*n* = 3; 10%). When asked to choose from a list of symptoms they were currently experiencing, the most commonly reported were fatigue (*n* = 25; 83%), cognitive challenges (*n* = 18; 60%), and peripheral neuropathy (*n* = 17; 57)^6^. Although not an objective of this pilot study, visual inspection of available data suggests those who took part in the yoga intervention only (*n* = 62) did not differ from study participants (*n* = 30) on age or diagnoses.

**Table 2:** Personal and medical characteristics of participants

	Yoga intervention participants (only)^§^	Study participants
Personal factors		
Age^†^	33.84 (6.06)	34.20 (5.09)
Location (*n*)		
Alberta	42	16
British Columbia	09	02
Manitoba	05	01
New Brunswick	01	01
Newfoundland and Labrador	04	01
Northwest Territories	01	00
Nova Scotia	02	00
Nunavut	00	00
Ontario	19	06
Prince Edward Island	00	00
Quebec	04	00
Saskatchewan	05	03
Yukon	00	00
Setting (*n*)		
Urban location (defined as > 100,000 people)	–	24
Rural/remote location (defined as all non-urban locations)	–	06
Biological sex (*n*)		
Female	–	28
Male	–	02
Current gender (*n*)		
Female	–	27
Male	–	02
Others	–	01
Marital status (*n*)		
Never married	–	10
Married	–	14
Common law	–	06
Education (highest level attained) (*n*)		
Some university/college	–	02
Completed university/college	–	19
Some graduate school	–	03
Completed some graduate school	–	06
Annual income		
< $20,000	–	01
Between $20,000 and $39,000	–	02
Between $40,000 and $59,000	–	03
Between $60,000 and $79,000	–	05
Between $80,000 and $99,000	–	07
> $100,000	–	12
Employment status (*n*)		
Disability	–	11
Part-time	–	09
Homemaker	–	01
Full-time	–	08
Temporarily unemployed	–	01
Ethnicity (*n*)^‡^		
British	–	15
Western European	–	17
Eastern European	–	08
Northern European	–	04
Southern European	–	01
Aboriginal	–	01
East and Southern Asia	–	02
Southern Asia	–	01
Western Asia	–	00
Pacific Islands	–	01
Arab	–	00
Latin/Central and South America	–	01
Caribbean	–	00
African	–	00
Others	–	
Medical factors		
Diagnosis (*n*)^‡^		
Blood	18	09
Breast	34	13
Digestive	07	00
Genitourinary	01	00
Gynecological	03	01
Head and neck	04	01
Lung	02	01
Metastases	–	08
Neurological	02	02
Others	03	01
Skin	01	01
Thyroid	06	02
Treatment status (*n*)		
On-treatment	–	14
Off-treatment	–	13
On-treatment (current treatment) (*n*)		
Surgery	–	0
Chemotherapy	–	4
Radiation	–	00
Hormone therapy	–	11
Biological therapy	–	01
Others	–	02
On-treatment (completed treatment) (*n*)		
Surgery	–	13
Chemotherapy	–	11
Radiation	–	09
Hormone therapy	–	01
Biological therapy	–	01
Others	–	03
Off-treatment (completed treatment) (*n*)		
Surgery	–	07
Chemotherapy	–	07
Radiation	–	05
Hormone therapy	–	00
Biological therapy	–	00
Others	–	02
Diagnosis date		
1990–1995	01	00
1995–2000	00	00
2001–2005	01	00
2006–2010	02	00
2011–2015	12	05
2016–2020	74	25
Not provided	02	00
Symptoms (*n*)^‡ ^		
Fatigue	–	25
Pain	–	16
Lymphedema (swelling)	–	05
Peripheral neuropathy (tingling, numbness) or other nerve damages	–	17
Osteoporosis or bone loss	–	02
Ostomy (colostomy, ileostomy, J-pouch, urostomy)	–	00
Cognitive challenges (learning or memory problems, chemo brain, brain fog)	–	18
Weight maintenance issues	–	13
Breathing issues	–	06
Heart issues	–	02
Communication issues (speaking or hearing)	–	02
Others	–	02

“–” indicates that data were not collected

^§^Young adults affected by cancer at baseline (week 0), who had completed at least one session

^†^Mean (*SD*)

^‡^Participants could select 'all that apply'

### Feasibility and acceptability

#### Feasibility

**Fig. 1:**
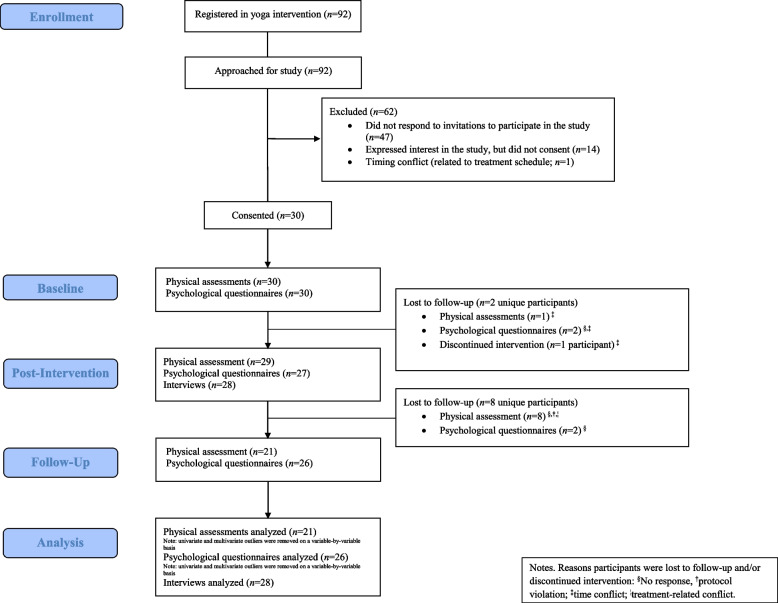
CONSORT flow diagram

Ninety-two young adults registered in the yoga intervention. All were invited to this study, 45 expressed interest, and 30 young adults completed informed consent and participated. Thus, the recruitment rate was 33%, which was below the a priori target of 60% (see Fig. 1).

With regard to participant retention to study procedures, of the 30 who consented to participate, 21 (70%) completed all physical assessments, online questionnaires, and interview according to the study schedule. This was below the a priori target of ≥ 75% of young adults in the study completing assessments on time. Of the nine participants who did not complete the scheduled assessments as intended, one dropped out and stopped attending the yoga intervention. When exploring retention to specific aspects of the study, 21 (70%) completed all scheduled physical assessments as intended, 26 (87%) completed all questionnaires capturing psychological outcomes, and 28 (93%) completed the interviews. For participants who completed the assessments as intended, there were < 5% missing data across physical assessments and psychological questionnaires (which met the a priori target of < 10% missing data). Based on Little’s Missing Completely at Random (MCAR) test, data were deemed missing at random (all *p*>0.05). For the interviews, there was one instance of missed questions due to the participant’s time restrictions.

Participants’ attendance to yoga classes varied from three (38%) to eight (100%) out of eight classes, with an average attendance rate of 6.40 (SD = 1.43) of eight classes (80%). Twenty-four out of 30 (80%) participants attended ≥ 75% (i.e., six or more of the eight classes), exceeding the a priori target (75% of participants would attend six or more classes). Reasons for missed classes included medical appointments (*n* = 3), scheduling conflicts/competing demands (*n* = 5), and not feeling well (*n* = 1). There were 41 unexplained absences despite follow-up emails to participants from the moderators. Of note, four participants attended more than one class per week. No adverse events were reported.

#### Acceptability

Table 3 includes category descriptions and representative participant quotes from the semi-structured interview questions that probed aspects of program acceptability. Participants shared that, in general, their expectations were met or exceeded in the yoga intervention. Participants appreciated that the yoga intervention was delivered via videoconference, which reduced some of their barriers to attending, and described the personnel delivering the intervention favorably. Indeed, the personnel were viewed as critical to fostering feelings of inclusivity and safety. Similarly, the nature of the yoga intervention, which focused on tailoring and modifying to meet each participant’s needs, was greatly appreciated. The additional class components were also deemed acceptable, with participants sharing how they enjoyed the class themes, music, behavior change support, and journaling and reflection.

**Table 3:** Selected representative quotes from participants

Category label	Exemplar quotes
**For the most part, expectations were met and exceeded.** This category captures when participants shared if (and how) their expectations of the yoga intervention, as a whole, were met, changed, or exceeded. Within this category, participants shared how their expectations changed over time. For example, participants did not initially expect there to be reflection/journaling yet came to expect and value this aspect of the intervention. As another example, participants shared that they had expectations about the intensity of the intervention, initially feeling as though it would be more challenging, but came to value the lighter intensity. Regardless of the initial expectations for the yoga intervention (as a whole), participants were of similar perspective that any expectations they did have were met and surpassed. Of note, there was one aspect of the intervention that did not meet the participants’ expectations: the group-based format. Participants shared that they expected to feel more connected with the other young adults affected by cancer who were taking part. Participants acknowledged that the lack of connection they felt may have been influenced by the nature of the online classes wherein only the instructor was visible throughout the class and participants had the option to practice with their cameras on or off. Participants offered some suggestions to try to improve feelings of connection, which are shared below in the category: **More could be done to promote connection in this group-based intervention.**	*“I didn’t necessarily feel connected. It’s so hard to, and you don’t really know the other people, so I just felt like I was doing it myself, on my own, with the instructor there as a third party. So, I don’t necessarily feel connected to the group, but I did look forward to it.”* YA1 *“With the reflection piece after and the [journal prompts], it sparked that in me a little bit, it changed the expectations a bit about not just being a yoga class but being something to take throughout my week too.”* YA3 *“I don’t really go into things with much expectation. I knew it was going to be good. I didn’t know it was going to be great. Like, it was awesome. I go into things without much expectation, but it would have exceeded the little expectation I had. I just wanted to be a part of it, and it was really awesome.”* YA10 *“I don’t know if it’s a good thing or not, everybody having their cameras off. […] I always turn my camera off, pretty much because most other people did it, and also because it’s kind of nice. Especially for me working from home, I’m on a screen all day long. So it’s kind of nice to have that option. But then at the same time I felt like you don’t really get to connect with the other people.”* YA24 *“I was hoping for more of a workout, and maybe I didn’t come in thinking it was what it was intended to be. But anyways it was super beneficial. Whatever the intention was, it was beneficial for that. I just would happily [like to see the classes] a step up in hardness if you will.”* YA28 *“I didn’t really feel like the sense of community [was something] that I wanted with the other participants. [Since] it was optional to have the camera on, and also I just had my screen on like speaker mode […]. Also, it’s not about everyone else. Like, it did feel like I wasn’t alone, but I didn’t feel like that sense of like there’s someone right here besides me that’s like breathing with me, that kind of thing. […] I know it’s not mandatory and you can’t force people, and has nothing to do with you guys or the program, but it would have been nice to hear more from the rest of the participants because not everyone wanted to share or felt comfortable or sometimes I didn’t have time to read through [the chat] instead of listen, and myself I also hadn’t spoke sometimes too so that might be my fault too.”* YA34 *“It’s been a great experience. There are a lot of things I really appreciated about it. I got more out of it than I expected, which was great. […] I did not expect to do the journaling, reflective bit at the end. I was like ‘no, I’ll just do the yoga and go’. [I was like] ‘yeah, yeah, uh huh, sure cool’, but some of the reflective questions were very, very important to me. So, I found myself writing them down and I still have them.”* YA36
**The videoconference-based delivery style enhanced accessibility.** This category captures when participants shared their appreciation for the videoconference-based delivery style of the yoga intervention. Within this category, participants shared how the videoconference-based delivery style reduced, and in some cases eliminated, barriers to attending (e.g., low/no cost, driving time). They also described how it offered them access to an intervention that they might not otherwise have (i.e., living in different cities/provinces, residing in rural and remote areas) and appreciated being able to do it from the comfort of their own home. Within this category, participants also shared how they liked having the option to choose from various times of classes when they signed up.	*“I liked the Zoom access, I liked that I could be [at home] and you guys were running this in Calgary. I never would have [been able to participate otherwise].”* YA1 *“[The fact that it] was online that you could it from home, it made it easy and accessible, it was at a very good time too.”* YA3 *“I’m in the middle of nowhere. We’re a long way from a lot of places so, it’s nice to be able to do something again, and it’s nice to have similar aged people [to practice with].”* YA11 *“I think you guys made it very accessible. Having it multiple times throughout different times of day was [also] really nice.”* YA13 *“[Having access to online] free classes [like yoga] rather than in-person were [something] I was considering for a while. […] [These classes] are nicer than [doing them from] YouTube.”* YA22 *“The fact that it was free was a huge incentive.”* YA24 *“[I liked that it was] in your own house and you didn’t have to drive somewhere. It was handy because it was just in your house and you did just have to [go out and buy equipment/props], it wasn’t like it was anything crazy. I did feel like it was very accessible to anybody so that was really probably made it a better option honestly for cancer people.”* YA33
**The personnel fostered feelings of inclusivity and safety.** This category captures when participants expressed feelings of gratitude towards the personnel delivering the yoga intervention. The personnel were viewed by participants as fostering a safe space that was welcoming of individuals with different cancer diagnoses and abilities. The instructors were described kindly and as important to promote a sense of relaxation and ease. Although some participants did not interact directly with the moderators, their presence and availability were viewed favorably. Participants saw the instructor and moderators as dynamically and effectively managing class needs and offering a safe and inclusive space.	*“I think just the personality of the instructor, they just created a safe and easy space [to practice yoga], it felt comfortable right away and non-threatening and there was never any pressure.”* YA3 *“[The yoga instructor] offered this safe space […] for myself to recuperate and regenerate and reset my day and take the time to relax.”* YA6 *“I think it was probably good to have [the moderators] there because I think you need to have someone [else other than the instructor] there. I never realized them but it was probably nice to have someone there if you did have a question or if you needed to get something it was probably nice to have that extra support as well for [the instructor as well].”* YA12 *“You’re going to have three people [one instructor and two moderators] assigned to the class for, [however many] people that would attend—but actually, you need those three people there because of [various reasons]. […] I’m sure they’re also allowing people to enter [the class]. So, no, definitely the number was appropriate, and all their roles were very appropriate.”* YA21 *“[The yoga instructor was] very personable, very easy to connect to. Whenever you arrived [to class], she had a big smile. She was very good at pulling everybody in. I think [she] did a really good job of just making it feel like, I think there were 18 people or something in my class, so she did a good job with just making it feel like she was talking to us.”* YA28 *“I think with the combination of the three of you working there behind the scenes, whether it’s like answering questions in the chat or um showing options while the instructor was teaching I think like I thought it was awesome. Like, all grounds were covered.”* YA31 *“[The yoga instructor was] so empathetic and gentle. […] She seemed very genuine and kind, and it made it a lot easier to just kind of relax and do what I was there to do.”* YA36
**The modifiable and personalized nature of the intervention was appreciated.** This category captures when participants expressed satisfaction with the modifiable and personalized nature of the yoga intervention. Participants appreciated being provided options within each posture to support their needs and abilities (whether looking for more/less challenging postures). Participants also described how they appreciated the verbal cues and variety of pose demonstrations, including seated and chair options (though many did not utilize the chair modifications, they recognized other participants may have needed them).	*“I really liked how the instructor was offering different variations of the poses we were doing. So, if there were some days when I was not feeling well and could not do everything, I could do something else. […] Normally if you do yoga with a group, everybody is like okay now we do this, and then everybody does this, that’s not super practical in this situation. So, having that opportunity to be like okay, it’s okay if you can’t do this, if you are not feeling it, you can do this instead, I think that really helps to keep the feeling like you are part of the group and feeling like being engaged in, I can do something as well, even If I can’t do like the hardest thing.”* YA6 *“I [really] liked being introduced to movements that I could do comfortably.”* YA9 *“I liked that [the poses were] easy to do. And that [the instructor] did the modifications, saying ‘OK, if you can do this, then you can try doing this as it’s a little further’ so, I really liked the modifications, and that kind of is the baseline and if you can do more here, do more. So, I felt that was nice and it kind of gave you an option.”* YA11 *“I feel like the instructor […] tailored things when necessary. The approach was very inclusive to varying and different skill levels or abilities or even just the kind of day somebody was having.”* YA15 *“I really enjoyed going to [the] classes. I wouldn’t use the other seated [modifications] and I didn’t use those but I thought it was cool that they were there [for others to potentially use].”* YA29 *“… I liked that [I was] introduced to movements that I could do very comfortably. […] Having access to things that you can do you comfortably, that aren’t too tiring, that you’re like ‘OK, this isn’t going to hurt me,’ and that add movement to your day [was valuable].”* YA39
**The additional class components were highly valued.** This category captures participants’ thoughts on the additional class components (e.g., class theme, poses, music, journaling, and reflection). Participants expressed satisfaction and deemed additional class components to be acceptable in general. Specifically, participants enjoyed the class themes (physical focus and energetic intention), which were embedded throughout the physical poses, behavior change components, music, and journaling and reflection. However, participants were of mixed minds about the journal prompts. Though all appreciated the opportunity to reflect and (in some cases) discuss the prompts within the 1-h class, others desired this aspect of class to be shorter or outside of the 1-h class time. This feedback was provided by participants to optimize delivery and allocate more time to practice yoga.	*“It was a good program. Again, I feel like it was a really good mix of restoration and more strength stuff. I liked the mix of that. I liked the journaling aspect at the end of each practice. I loved that they sent the Spotify playlist, I felt that was really fantastic and it took me a few weeks to be like, “Oh yeah the Spotify!” and figure out how to run that in the background while also doing the program but once I did I was like, oh this is a really nice touch, I really like that.”* YA1 *“I loved the combination of [the behaviour change support, the mindfulness meditation, and the physical postures], I think they did a very good job of putting that together too like as I said, [I liked how] themes each week would go together with the readings, and the music, those practices we did too.”* YA6 *“I think setting an intention before the practice was really helpful cause for me to set the intention before the practice I could decide what my goal was and I would preemptively pick something that wasn’t particularly physical and just about re-connecting with myself or whatever that prompt was, it allowed me to remember what I was practicing that day.”* YA13 *“I loved the playlist actually I think that that was a really unique way for everybody to experience the same thing but not necessarily like directly from the instructor. So if there was a particular piece of music that didn’t quite resonate with you or didn’t quite resonate with the movement that you were doing at the time it was very easy to kind of move to the next song. I just thought that that was unusual compared to some of the other yoga experiences that I’ve had.”* YA15 *“[I would suggest] a shorter amount of time to interact with the prompts, because I think the prompts and reflection was super helpful, but it was like 15 minutes, and I think that was maybe too long.”* YA38
**Access to a greater frequency of classes per week was desired.** This category captures participants’ desire for more yoga classes to be offered throughout the week. Participants shared that although they appreciated having access to one class each week; if they had to miss class that week, they missed their opportunity to practice yoga, which they did not like. Suggestions were made to have the intervention include two classes per week. Although recognizing that offering classes twice per week may present more scheduling challenges, participants felt that increasing the frequency of classes would allow them to accumulate more benefits from a regular and consistent practice.	*“[The yoga intervention] was run really great, if [classes were] offered a few times a week I would be up for that too.”* YA3 *“I think personally I would have enjoyed like twice a week, I think that would be super helpful not just taking the time once a week to slow it down but If I could do it twice a week, I could re-orient myself earlier in the week and work towards the goals I need.”* YA6 *“I think anything more would just—I mean I would love to go twice a week, but I think that would just be a lot of work for you guys.”* YA10 *“I’d like to do it twice a week, but I don’t know in terms of scheduling for me personally how that would work but it’s something for me that like twice a week would be helpful.”* YA11 *“I think once a week was good, but I think twice a week would probably make a bigger difference for me. Personally, just I think with increased frequency I would probably notice a little bit more differences in some of the other some of the aspects that we talked about.”* YA15 *“I think twice [a week] is better. One time was fine, but I do think twice a week would be really beneficial. […] I think you just get yourself into even more of a groove [with 2 times a week], because if you missed one yoga class, like that’s huge, but if you miss one [out of 2] classes it is not quite so huge.”* YA28 *“I think in order for yoga to be really beneficial, you need to be doing it more often. So, I think twice a week would be even better. Once a week was good and it was like oh feel so good to stretch out and then if you don’t make a conscious effort to do it again on your own, it feels like you’re not seeing many bigger improvements, right? I think that you’d see more [improvements], or for me at least, I’d see more flexibility and like stuff like that if I did it more regularly.”* YA38
**More could be done to promote connection in this group-based intervention.** This category captures when participants shared their suggestions to improve the group-based nature of the intervention. While participants commented that the young adult-specific nature of the intervention was appreciated and they liked being around similar others, they did not feel connected. Though this lack of connection was described as inconsequential and having minimal impact on their enjoyment of the yoga intervention, participants felt the intervention could be optimized by leveraging the social aspect and offered some suggestions to do so (e.g., introductions and icebreakers during the first few classes).	*“I have really enjoyed that hour of my day once a week. I don’t necessarily feel connected [to other participants] because it’s so hard to and you don’t really know the other people so I just kind of feel like I’m doing it myself on my own with the instructor there as a third party so, I don’t necessarily feel connected to the group, but I do look forward to it.”* YA1 *“Our group did most of the communication over chat, and I guess it would have been nice if we did more conversation, which is kind of hard to do over Zoom when you don’t know people. But yeah, if we could have created a bit more of that.”* YA3 *“I do wonder what the options would be for other interactions whether it’s adding in a more official sense, adding an additional 10 or 15 minutes at the beginning or something like that, the expectation is that you show up, not just like you have the option, but all show up at this time, we’re going to start with a grounding exercise and a quick go round or something like that, and then get into the program as a way of facilitating that sense of belonging and mutual support.”* YA8 *“I would have liked to see a bit more of an introduction to the group members to create a bit more of a relationship between the members. […] It might be nice to connect on a slightly different level. So you know a kind of ice breaker, maybe a session that’s not yoga, and then the following week you move into a yoga piece. […] I really enjoy being able to share something with other cancer survivors or other cancer patients that are very similar and even though we were, you know from across the country. It was this one period of time where we got to kind of all be together.”* YA15 *“I don’t really feel like I know people on a super personal level, but it has been nice to see the same people every week and everyone is quite friendly and supportive. So, I guess it feels like there’s just sort of a nice place that you can join once or twice a week. Even if you don’t really know anyone, I feel welcome and that it’s gonna be nice activity to do that there’s a good, positive attitude coming out from everyone.”* YA39

Though high levels of satisfaction and acceptability were expressed, participants also raised several points of consideration for improving the yoga intervention. Despite having the option to attend more than one class per week, participants commented that they would have preferred this to be built into the structure of the intervention, and that a greater choice (days/week) and flexibility would be appreciated. Beyond this, participants described appreciating the group-based nature of the program but commented that they did not feel connected to their group. Participants in this sample would have appreciated the opportunity to connect more or to other participants and suggested activities and introductions among intervention participants as a way to foster greater connection. Finally, while some participants valued the reflection and behavioral support offered near the end of class, others described a preference for these components to be offered closer to the beginning to facilitate their ability to integrate during class time. No issues relating to study methods were identified and the number, timing, and duration of assessments and study-related procedures were deemed acceptable. Participants reported being grateful to receive yoga and were happy to contribute to the research.

### Implementation

Six moderators completed 6 h of study-specific training. This training time excluded their 12-15 h Thrive Health Exercise Oncology, 8 h Thrive Center, and 3 h general moderator training. Three yoga instructors completed 8 h of study-specific training. This training time excluded their pre-existing minimum 200-h yoga teacher training, 12–15-h Thrive Health Exercise Oncology training, and a minimum of 30 h completing their Yoga Thrive teacher training (or equivalent). With regard to delivery resources, each 8-week yoga session constituted 12 delivery hours (one class/week for a 60-min yoga class plus a minimum of 15 min before and after each class), wherein two moderators and one instructor were present (totaling 36 personnel hours). Between fall 2020 and winter 2021, five yoga sessions were provided (fall 2020 *n* = 2 sessions; winter 2021 *n* = 3 sessions). Thus, there was a total of 180 delivery hours. Physical assessments and interviews took a total of 55 h for research staff (who were also trained moderators). Administrative support summed to an additional 20 h outside of intervention delivery and assessment/interview time, including tasks such as sending email reminders and scheduling assessments.

In terms of the fidelity checklist completed by moderators, there were no instances of deviation across instructors and classes in terms of greeting and closing classes, offering modifications, and using autonomy-supportive language. For the yoga instructors, fidelity checks over the two waves (fall 2020/winter 2021) and five sessions indicated a total of six recorded instances of posture omission (due to time constraints) and 23 instances of a posture being taught at another time in class (i.e., before or after it was intended) out of the 186 postures covered within the 8-week yoga intervention.

### Potential effects of yoga

#### Physical outcomes

Participants’ scores on physical assessments at baseline varied from within normal ranges to poor (or low), see Table 4. Specifically, scores on balance and shoulder range of motion were within normal ranges, whereas scores for functional mobility and flexibility were below normative age-related values. With regard to changes, there were large, significant changes in functional mobility (i.e., sit-to-stand test; *F*(2, 40) = 8.261, *p ≤* 0.001, $\eta_{p}{}^{2}=0.292$) and flexibility (i.e., sit and reach test) on both the right (*F*(2, 40) = 3.959, *p* = 0.027, $\eta_{p}{}^{2}=0.165$) and left sides over time (*F*(2, 40) = 3.524, *p* = 0.039, $\eta_{p}{}^{2}=0.150$), indicative of improvements in these outcomes. There were no significant differences observed over time (baseline, post-intervention, follow-up) on physical assessments of balance or shoulder range of motion (all *p*>0.05), and effect sizes ranged from small to medium ($\eta_{p}{}^{2}s=0.048-0.098$).

**Table 4:** Potential effects of the yoga intervention

Outcomes	Scale range	Mean score (SD)			*F*-statistic	*p*-value	Effect size (*η*_*p*_^2^)
		Baseline (week 0)	Post-intervention (week 8)	Follow-up (week 16)			
Physical							
Single leg balance (s)	–						
Right (*n* = 19)^¡,•^	0–45	43.46 (6.01)	45.00 (0.00)	43.33 (5.11)	1.313	0.281^‡^	0.068
Left (*n* = 20)^¡,•^	0–45	43.69 (4.23)	42.96 (6.65)	44.36 (2.86)	2.056	0.167^†,‡^	0.098
Sit and reach (cm)	–						
Right (*n* = 21)	–	− 3.14 (14.62)	3.36 (17.86)	2.69 (15.78)	3.959	0.027^‡^	0.165
Left (*n* = 21)	–	− 2.57 (14.17)	2.38 (17.93)	3.02 (16.05)	3.524	0.039	0.150
Shoulder range of motion (°)	–						
Right (*n* = 21)	–	162.95 (10.13)	165.17 (11.18)	165.36 (9.99)	0.997	0.348^†^	0.048
Left (*n* = 21)	–	161.88 (12.60)	163.38 (13.94)	164.40 (12.04)	1.328	0.274^†,‡^	0.062
30-s sit-to-stand (reps) (*n* =21)	–	14.14 (4.35)	17.19 (5.31)	17.33 (4.71)	8.261	<0.001	0.292
Psychosocial							
RAND-36							
Physical functioning (*n* = 26)	0–100	75.38 (23.41)	77.41 (23.37)	79.04 (18.22)	0.489	0.545^†,‡^	0.019
Role limitation due to physical health (*n* = 24)^§^	0–100	54.17 (37.35)	51.39 (37.00)	46.88 (42.55)	0.577	0.566^‡^	0.024
Role limitation due to emotional problems (*n* = 26)	0–100	51.28 (39.14)	48.72 (39.14)	52.56 (43.38)	0.121	0.886^‡^	0.005
Energy/fatigue (*n* = 26)	0–100	35.32 (18.13)	40.38 (15.29)	43.08 (14.00)	3.523	0.037	0.124
Emotional well-being (*n* = 26)	0–100	60.69 (20.25)	65.38 (18.62)	67.62 (17.25)	2.810	0.070	0.101
Social functioning (*n* = 26)	0–100	63.46 (20.89)	71.15 (22.01)	74.52 (19.52)	3.894	0.027^‡^	0.135
Pain (*n* = 25)^¡^	0–100	74.00 (15.17)	73.50 (18.51)	71.80 (15.28)	0.263	0.770^‡^	0.011
General health (*n* = 26)	0–100	51.15 (21.37)	48.65 (18.84)	51.92 (21.78)	0.844	0.405^†^	0.033
FACIT-Fatigue (*n* = 26)	0–52	30.73 (9.00)	32.50 (9.76)	33.81 (7.92)	2.608	0.097^†^	0.094
Brief Resilience Scale (*n* = 25)^§^	1–5	3.28 (0.93)	3.36 (0.76)	2.97 (0.23)	3.012	0.074^†,‡^	0.111
Posttraumatic Growth Inventory (*n* = 25)^§^	0–105	58.48 (22.14)	54.48 (20.03)	55.40 (23.62)	1.626	0.207	0.063
MBSRQ-AS							
Appearance evaluation (*n* = 25)^§^	1–5	2.85 (0.79)	2.79 (0.87)	3.03 (0.84)	3.918	0.036^†^	0.140
Appearance orientation (*n* = 25)^§^	1–5	3.01 (0.63)	2.91 (0.61)	2.94 (0.65)	1.275	0.289	0.050
Body areas satisfaction scale (*n* = 25)^§^	1–5	3.08 (0.58)	3.00 (0.67)	3.03 (0.64)	1.011	0.372	0.040
Overweight preoccupation (*n* = 25)^§^	1–5	2.25 (1.04)	2.27 (0.96)	2.45 (1.10)	2.436	0.098^‡^	0.092
Self-classified weight (*n* = 25)^§^	1–5	3.42 (0.99)	3.30 (0.91)	3.28 (1.00)	3.108	0.054	0.115
Five Facet Mindfulness Questionnaire							
Observing (*n* = 24)^§,¡^	8–40	25.75 (3.85)	24.63 (4.92)	25.33 (5.01)	1.238	0.299^‡^	0.051
Describing (*n* = 25)^§^	8–40	24.12 (5.37)	24.56 (6.25)	24.60 (6.44)	0.410	0.666	0.017
Acting with awareness (*n* = 25)^§^	8–40	24.48 (7.18)	23.76 (6.99)	23.80 (6.96)	0.737	0.484	0.030
Non-judging of inner experience (*n* = 25)^§^	8–40	25.48 (7.74)	25.80 (7.57)	26.28 (8.40)	0.273	0.762	0.011
Non-reactivity to inner experience (*n* = 25)^§^	7–35	18.72 (4.65)	20.24 (4.33)	20.32 (4.52)	3.922	0.026^‡^	0.140
10-Item Perceived Stress Scale (*n* = 25)^§^	0–40	20.64 (7.07)	19.36 (7.12)	17.80 (6.68)	4.912	0.011	0.170
Group Identification Scale (*n* = 26)	1–7	–	4.91 (1.00)	–		–	–

*FACIT* functional assessment of chronic illness therapy, *MBSRQ-AS* Multidimensional Body-Self Relations Questionnaire-Appearance Scales, *reps* repetitions, *SD* standard deviation

^§^A participant was missing data at one or more time points

^†^Sphericity has been violated; in these cases, the Greenhouse-Giesser correction was used to inform the *p*-values and effects sizes

^‡^Shapiro-Wilk test of normality was significant at one or more time points

^¡^One (or more) univariate outlier(s) was (were) removed

^•^One (or more) multivariate outlier(s) was (were) removed

#### Psychological outcomes

As seen in Table 4, participants’ scores at baseline on the psychological questionnaires were low relative to scale ranges, indicative of generally poorer quality of life; worse symptoms of fatigue; low amounts of resilience, posttraumatic growth, body image, and mindfulness; and moderate levels of stress. There were significant changes over time with medium to large effect size on participants’ quality of life (subscales of energy/fatigue [*F*(2, 50) = 3.523, *p* = 0.37, $\eta_{p}{}^{2}=0.124$] and social functioning [*F*(2, 50) = 3.894, *p* = 0.027, $\eta_{p}{}^{2}=0.135$]) body image (subscale of appearance evaluation [*F*(1.619, 38.850) = 3.198, *p* = 0.036, $\eta_{p}{}^{2}=0.140$]), mindfulness (subscale of non-reactivity to inner experience [*F*(2, 48) = 3.922, *p* = 0.026, $\eta_{p}{}^{2}=0.140$]), and stress ([*F*(2, 48) = 4.912, *p* = 0.011, $\eta_{p}{}^{2}=0.170$)]), indicative of improvements in each of these outcomes. There were no significant differences over time on the remaining psychological outcomes (all *p*>0.05), with effect sizes ranging from small to medium ($\eta_{p}{}^{2}s=0.005-0.115$). Finally, participant’s score on group identification, measured post-intervention only (mean = 4.91; SD = 1.00), indicated that participants did not identify with their yoga group.

## Discussion

The purpose of this single-arm hybrid effectiveness-implementation pilot study was to better understand feasibility, perceptions of acceptability, implementation, and potential effects of yoga delivered via videoconference to young adults affected by cancer. Although recruitment and retention to the study was lower than has been reported elsewhere [22, 23], for those completing the study, attendance rates were high and missing data was low. Participants were generally satisfied with the intervention and found the intervention and study methods acceptable. Importantly, participants also provided recommendations to improve the intervention. Overall, these findings provide early evidence supporting the potential benefits associated with yoga among young adults affected by cancer and highlight critical modifications to study and intervention components that support gathering further evidence for the effectiveness and implementation of yoga delivered via videoconference for this cohort.

Despite relatively large numbers within the yoga intervention (*n* = 92), recruitment to the study was low (*n* = 30, out of *n* = 45 interested). It is possible that many in this cohort simply did not wish to take part in research, which has been described elsewhere [54]. It is also possible that the additional time required for the study assessments was a deterrent to young adults who already manage a number of competing work/life demands [18, 19]. Indeed, retention to assessments was lower than has been reported previously [55, 56], which could be reflective of scheduling conflicts, burdensome assessments, or lack of interest to complete the assessments, though this was not mentioned in the interviews. Finding ways to maximize scheduling flexibility for participants and reducing study-related barriers should be explored, but this must be balanced alongside the practical considerations of conducting a study (e.g., personnel schedules). Another strategy that may enhance recruitment and adherence to assessments could be sharing videos that show what the assessments involve so participants can have a clear idea of what is required.

Notwithstanding the lower than anticipated recruitment and retention rates, nearly all participants remained engaged in the yoga intervention and attendance was higher than anticipated [22, 23]. Participants in this sample reported valuing the nature of the intervention and the opportunity to engage online. These findings extend prior work wherein young adults have expressed satisfaction and appreciation for young adult-specific supportive care opportunities [22]. Aligning this intervention with young adults’ delivery style [20, 21] and activity preferences [57] was seemingly well-received. Looking ahead, providing opportunities for modification, integrating autonomy-supportive strategies, and continuing to deliver online may be critically important when offering yoga to young adults affected by cancer.

Beyond sharing aspects of the intervention that were appreciated, participants in this sample also offered several useful considerations for improvement, including offering the intervention at a greater frequency (e.g., 2 times/week) and integrating reflection and behavioral support throughout the practice, versus at the end of each class. This feedback has been incorporated, and Yoga for Young Adults Affected by Cancer is now a 12-week intervention (to support behavior change), offered 2 times/week, and it is being evaluated via a full-scale mixed-methods, hybrid effectiveness-implementation study (clinicaltrials.gov identifier: NCT05314803).

In terms of implementation, the study team devoted substantial time and resources to ensuring adequately prepared yoga instructors and moderators. The importance of well-trained personnel when delivering physical activity for individuals living with and beyond cancer has been iterated upon elsewhere [58] and is critical to ensuring safety and fidelity. In terms of the assessments, collecting quantitative (objective and self-reported) and qualitative data within this study afforded deeper insights into aspects of feasibility, acceptability, and implementation and may aide in gaining a more comprehensive understanding of the role of yoga within the young adult cancer experience. Nevertheless, considering ways to reduce the resources required, at both research and intervention implementation levels, may be warranted. Conducting assessments with multiple participants at the same time could be explored. However, this would need to be balanced with participant safety, comfort, and scheduling logistics, as well as the assessors’ ability to accurately administer the assessments. Finally, although the administrative time for this study was relatively low—likely due to shared responsibilities across study team members and email templates—processes have been further streamlined (e.g., using automated reminders via REDCap and an online scheduling system for physical assessments and interviews) and are being implemented in the full-scale study that is currently underway (see above referenced clinicaltrials.gov identifier: NCT05314803).

With regard to the potential effects of yoga, findings suggest that this yoga intervention may augment participants’ functional mobility, flexibility, aspects of quality of life (energy/fatigue, social functioning), body image (appearance evaluation), and mindfulness (non-reactivity to inner experience), while lowering perceived stress. These findings are similar to what has been reported previously in the adult cancer yoga literature [59–61]. Furthermore, while there were no statistically significant changes in the remaining physical and psychological outcomes, some (e.g., quality of life subscales of role limitations due to physical health and emotional well-being and the fatigue measure) met the cutoffs for minimal clinically important differences (MCIDs) [62–64]. For outcomes that were non-significant and that did not meet MCIDs, it is possible that findings are due to the small sample and pilot data collected or that yoga does not impact this outcome for this cohort. Looking ahead, identifying additional meaningful outcomes for this cohort will be important and is being explored in the full-scale study (see above) and in forthcoming work.

Notably, group identification, an important factor promoting adherence and supporting behavior change [50], was low in this sample. This was a somewhat surprising finding as young adults affected by cancer have reported social isolation in the context of COVID-19 [65, 66] and desiring opportunities to connect with other young adults [67]. Simply bringing young adults together to participate in a yoga intervention may not be enough to promote group identification. Including strategies within the yoga classes (e.g., icebreaker questions, breakout rooms) to facilitate the connection between participants was suggested by participants as a possible way to promote group identification (and has been incorporated in the full-scale study described above). However, it is also plausible that the online nature of the intervention may not be conducive to facilitating the depth of social support that young adults expected or desired. Exploring the type of connection desired, how to foster connection online, and how to determine what “connection” means for this cohort will be necessary for future work.

When interpreting the results from this pilot study, there are several considerations to keep in mind. First, participants did not always have the same individual performing their physical assessments, which may have reduced the reliability of the assessment and results. Efforts were taken to mitigate this through extensive training for moderators and protocol documentation. Second, the physical assessments were performed via videoconference and thus may reflect more variability than similar assessments conducted in person. Ongoing work is examining assessments conducted virtually [68]. Also, participants had varied equipment (e.g., chair heights, measuring tools) available to them at home. Though every effort was made to document instances of assessment modification based on participants’ home settings, this could have also impacted the results. Third, there were 14 instances of protocol violations (i.e., assessments conducted > week 2) at the baseline (week 0) assessment for the 14 participants who registered in the fall 2020 wave. This was due to the timing of receiving ethical approval to recruit to the study after the yoga intervention had launched. Though these violations could contribute to washing-out effects, the decision was made to retain these participants’ data given the nature of this pilot study. Fourth, the sample described herein was predominantly female, residing in urban locations, and likely represents highly motivated young adults who are both interested in yoga and keen to contribute to research. Looking ahead, exploring strategies to minimize selection bias and recruit a more diverse sample will be important. Fifth, data were gathered from a single group; thus, it is possible that improvements noted herein were a function of time or other factors, unrelated to the yoga intervention. Relatedly, data were not nested and no statistical adjustments to the significance level were made despite running a number of tests, inflating type I error. This decision was made given the pilot nature of this work.

Taken together, findings suggest that an online yoga intervention delivered to young adults affected by cancer across Canada is potentially feasible and safe and may afford both physical and psychological benefits. However, important modifications to the intervention and study are required to enhance feasibility and acceptability. Supporting recruitment efforts by providing intervention and research details, offering greater scheduling flexibility, increasing the frequency of classes/week, and enhancing opportunities for participant interaction could improve feasibility and acceptability. This study provided invaluable data that has been used to refine the yoga intervention, underscoring the value of pilot studies, and represents an important step towards better understanding and promoting yoga to more young adults affected by cancer across Canada.

## Supplementary Information

**Additional file 1.** Relevant checklists.

**Additional file 2.** Semi-structured interview questions asked to explore perspections of acceptability.

**Additional file 3.** Description and scoring of physical assessments.

**Additional file 4.** Description and scoring of questionnaires used to assess psychological outcomes.

## Data Availability

The datasets used and/or analyzed during the current study are available from the corresponding author upon reasonable request.

## Footnotes

1 Offering the intervention (yoga) independently from the study was a choice informed by pragmatic considerations and relevant research. Ethics board approval timelines, the distressing nature of the COVID-19 pandemic (and need to offer supportive care in a timely fashion), and documented study recruitment challenges in this cohort all influenced the decision to offer the intervention and subsequently recruit into the study.

2 Support persons were not approached for participation in this study.

3 Young adults were allowed to “register” and participate in more than one session (i.e., attend more than one class/week).

4 Data were generally normally distributed. Instances where the assumption of normality was violated have been documented in Table 4.

5 This decision was purely exploratory, as pilot studies are not typically powered to assess the statistical significance but can provide data to inform sample size calculations for full-scale studies.

6 Does not sum to 100% because participants could select all symptoms that applied.
