# Supplementary material for: Exploring feasibility, perceptions of acceptability, and potential benefits of an 8-week yoga intervention delivered by videoconference for young adults affected by cancer: a single-arm hybrid effectiveness-implementation pilot study
Source: Pilot Feasibility Stud. 2023 Mar 10;9:37. doi: 10.1186/s40814-023-01244-y (PMC9999078; doi:10.1186/s40814-023-01244-y)
Supplement: Supplementary file 2 — Additional file 2. Semi-structured interview questions asked to explore perspections of acceptability. [file 40814_2023_1244_MOESM2_ESM.docx]

Supplementary File 2. Semi-structured interview questions asked to explore perspections of acceptability.

1. What were your reasons for deciding to participate in the yoga intervention?

PROBE: Can you describe what this experience has been like for you? Have you done yoga before? If yes, describe how this compares.

2. Based on your participation, can you share with us what elements of the yoga intervention you found most useful/beneficial?

PROBES: Why? How did this element help you? Can you give me an example? Did you find any elements that were not useful? Can you please explain?

3. What were your biggest challenges at the start of the yoga intervention?

PROBES: Is this/are those thing(s) still hard for you now? If you have noticed changes, how did they change? Why do you think they changed?

4. Did you experience any barriers to attending the yoga intervention? If so, what were they?

PROBES: Why? What would have helped you or made it easier to attend? Were there elements of the intervention that made it harder for you to participate or feel engaged?

5. What things made it easier for you to attend the yoga intervention?

PROBES: Why? Is there anything the intervention or trial could have offered to make it easier for you? Were there elements of the program that made it easier for you to participate or feel engaged?

6. Thinking about the yoga intervention, we are curious as to what you liked and didn’t like.

PROBES: What did you think about the frequency of delivery? Of select aspects of the intervention (e.g., mindfulness/meditation, physical postures)? Of the entire intervention? Of the instructor? Of the moderators? What would you like to see more or less of?

7. How did you feel about specific elements of the intervention; for example, the first 45-minutes of yoga? The last 15-minutes of mindfulness and discussion?

PROBES: Can you give me an example? Were there elements within each of these components that you liked or didn’t like? Why or why not? Which component did you find most engaging and why?

8. What did you think about the group format of the yoga intervention?

PROBES: What about the group format was important (or unimportant) to you?

9. When you first started, was there anything that you expected to gain by being in this yoga intervention?

PROBES: In what ways were those expectations met? In what ways were they not met?
