# Supplementary material for: Exploring feasibility, perceptions of acceptability, and potential benefits of an 8-week yoga intervention delivered by videoconference for young adults affected by cancer: a single-arm hybrid effectiveness-implementation pilot study
Source: Pilot Feasibility Stud. 2023 Mar 10;9:37. doi: 10.1186/s40814-023-01244-y (PMC9999078; doi:10.1186/s40814-023-01244-y)
Supplement: Supplementary file 3 — Additional file 3. Description and scoring of physical assessments. [file 40814_2023_1244_MOESM3_ESM.docx]

Supplementary File 3. Description and scoring of physical assessments.

| Outcome category | Name of assessment | Description of assessment | Notes and common modifications | Scoring (range) | Explanation of score range |
| --- | --- | --- | --- | --- | --- |
| Single-leg balance | Single Leg Balance Test | Participant was instructed to stand barefoot with hands on opposite shoulders, crossed in front of their chest. Once the participant was stable, the assessor asked the participant to stand on a leg of their choice, lifting the opposite foot (so it is near, but not touching the standing leg). Time was started once the participant lifted their foot off the ground, to a maximum of 45 seconds. If/when the participant lost their balance, within the initial 3 seconds (s) of the trial, a second trial was allowed. In this case, the better trial for each leg was recorded. This was repeated for the opposite leg. |  | 0-45s  *Recorded to the nearest 0.1s | Higher scores indicate greater balance. |
| Shoulder range of motion | Shoulder Range of Motion (ROM) Test (Shoulder Flexion) | Participant was instructed to place a chair perpendicular to the video screen. The participant was then instructed to sit tall, facing forward in the chair (i.e., their shoulder perpendicular to the video screen), with their feet flat on floor. The participant was then instructed to rotate their hand into a neutral position (i.e., thumb facing up) and to keep their elbow extended. When the participant was ready, they performed shoulder flexion as far as they could without compensation (e.g., arched back, bringing arm away from ear). Once the participant reached their full range of motion position, the assessor took a screenshot (i.e., picture) of the video screen. This was repeated for each arm twice and the average of both trials was used. |  | 0-180 degrees  *Recorded to the nearest 1 degree | Higher scores indicate greater shoulder range of motion. |
| Flexibility | Seated Sit and Reach Test | Participant was instructed to place a chair perpendicular to the video screen and sit on the edge of the chair, extending one leg out at a time. The participant was then instructed to grip a ruler/measuring tape with both hands, and lean forward to touch the toes of their extended leg. The knee of extended leg remained straight. The participant was instructed to hold the maximum flexion position for 2 seconds before recording.  The participant was then asked to measure the distance from the ruler/measuring tape device to the tip of their toes. They then stated the number they saw on the ruler/measuring tape to the assessor. This was repeated for each leg twice and the better score was used. | The sit and reach was to be completed sitting on a chair with a ruler to perform the measurements. Instances where a participant did not have a ruler, a measuring tape was used. The study team recorded measurements in centimeters (cm), so in the case where the participant’s measuring device recorded in inches, the study team made the conversion. | +/- distance from toes in cm  *Recorded distance from toes to fingertip to nearest 0.5 cm. | Lower negative scores and higher positive scores indicate greater hamstring flexibility. |
| Functional mobility | 30 Second Sit-to-Stand Test | The participant was instructed to sit on a chair with their on opposite shoulders in front of chest. The participant was to come to a full standing position, and then touch their glutes back to the chair (i.e., sit-to-stand) without hand assistance. The participant was instructed to complete as many sit-to-stands as possible in 30 s. This test was completed only once. | The sit-to-stand test was often completed with a standard kitchen chair (approximately 17 inches). Although there were some instances where a chair or stool was used that may have been higher or lower, based on the availability to the participant. In addition, the participant was encouraged to use the same chair each time to ensure consistency, however for pragmatic reasons, that was not always the case (e.g., when a participant completed the assessment in a different space, chair being occupied by someone else). | Number of repetitions completed in 30s | Higher scores indicate greater functional mobility. |
