# Supplementary material for: Exploring feasibility, perceptions of acceptability, and potential benefits of an 8-week yoga intervention delivered by videoconference for young adults affected by cancer: a single-arm hybrid effectiveness-implementation pilot study
Source: Pilot Feasibility Stud. 2023 Mar 10;9:37. doi: 10.1186/s40814-023-01244-y (PMC9999078; doi:10.1186/s40814-023-01244-y)
Supplement: Supplementary file 4 — Additional file 4. Description and scoring of questionnaires used to assess psychological outcomes. [file 40814_2023_1244_MOESM4_ESM.docx]

Supplementary File 4. Description and scoring of questionnaires used to assess psychological outcomes.

| Outcome  category | Name of questionnaire | Number of items | Scale range | Description for computing scores | Score range | Explanation of score range |
| --- | --- | --- | --- | --- | --- | --- |
| Quality of life | [RAND 36-Item Short Form Health Survey](https://www.rand.org/health-care/surveys_tools/mos/36-item-short-form/scoring.html) | 36 | Differs across items:  1 (*excellent*) to 5 (*poor*)  1 (*much better now than one year ago*) to 5 (*much worse now than a year ago*)  1 (*yes, limited a lot*) to 3 (*no, not limited at all*)  1 (*yes*) to 2 (*no*)  1 (*not at all*) to 5 (*extremely*)  1 (*none*) to 6 (*very severe*)  1 (*all of the time*) to 6 (*none of the time*)  1 (*definitely true*) to 5 (*definitely false*) | Scores were transformed according to instructions, and then items were summed on each subscale (i.e., physical functioning, role limitations due to physical health, role limitations due to emotional problems, energy/fatigue, emotional well-being, social functioning, pain, general health). | 0-100 | Higher scores indicate greater quality of life. |
| Fatigue | [FACIT-Fatigue Scale](https://www.facit.org/measures-scoring-downloads/facit-f-scoring-downloads) | 13 | 0 (*not at all*) to 4  (*very much*) | Items were summed, multiplied by 13, and then divided by the number of items answered. | 0-52 | Higher scores indicate lower fatigue. |
| Resilience | [Brief Resilience Scale](https://ogg.osu.edu/media/documents/MB%20Stream/Brief%20Resilience%20Scale.pdf) | 6 | 1 (*strongly disagree*) to 5 (*strongly agree*) | Items were summed, then divided by 6. | 1-5 | Higher scores indicate greater resilience. |
| Posttraumatic growth | [Posttraumatic Growth Inventory](https://www.careinnovations.org/wp-content/uploads/Post-Traumatic-Growth-Inventory.pdf) | 21 | 0 (*I did not experience this change as a result of my crisis*) to 5  (*I experienced this change to a very great degree as a result of my crisis*) | Items were summed.^†^ | 0-105 | Higher scores indicate greater growth. |
| Body image (appearance) | [Multidimensional Body-Self Relations Questionnaire Appearance Scales](https://digitalcommons.usf.edu/cgi/viewcontent.cgi?article=2076&context=etd) | 34 | Differs across items:  1 (*definitely disagree*) to 5 (*definitely agree*)  1 (*never*) to 5 (*very often*)  1 (*very underweight*) to 5 (*very overweight*)  1 (*very dissatisfied*) to 5 (*very satisfied*) | Averages of items on each subscale were computed (i.e., appearance evaluation, appearance orientation, body area satisfaction scale). | 1-5 | Higher scores indicate more positive body image. |
| Mindfulness | [Five Facet Mindfulness Questionnaire](http://ruthbaer.com/academics/FFMQ.pdf) | 39 | 1 (n*ever or very rarely true*) to 5 (*very often or always true*) | Items on each subscale were summed (e.g., observing, describing, acting with awareness, nonjudging of inner experience, nonreactivity to inner experience). | 8-40 (except for the non-reactive facet; 7-35) | Higher scores indicate greater mindfulness. |
| Perceived stress | [10-Item Perceived Stress Scale](https://www.sprc.org/system/files/private/event-training/Penn%20College%20-%20Perceived%20Stress%20Scale.pdf) | 10 | 0 (*never*) to 4 (*very often*) | Items were summed. | 0-40 | Higher scores indicate more perceived stress. |
| Group identification | [Group Identification Scale](http://goodmedicine.org.uk/sites/default/files/assessment%2C%20group%20identification%20scale.pdf)^‡^ | 4 | 1 (*strongly disagree*) to 7 (*strongly agree*) | Items were summed, then divided the by 4. | 1-7 | Scores < 5, indicates that the group is not identified with; scores > 5 indicates the group is identified with. |

Notes. ^†^No subscale scores were computed; ^‡^The questionnaires in this questionnaire were adapted to refer to the yoga group (i.e., I feel a bond with my yoga group).
